# Combined ChIP-Seq and transcriptome analysis identifies AP-1/JunD as a primary regulator of oxidative stress and IL-1β synthesis in macrophages

**DOI:** 10.1186/1471-2164-14-92

**Published:** 2013-02-11

**Authors:** Richard P Hull, Prashant K Srivastava, Zelpha D’Souza, Santosh S Atanur, Fatima Mechta-Grigoriou, Laurence Game, Enrico Petretto, H Terence Cook, Timothy J Aitman, Jacques Behmoaras

**Affiliations:** 1 MRC Clinical Sciences Centre, Imperial College London, Hammersmith hospital, Du Cane Road W12 0NN, London, UK; 2 Stress and Cancer Laboratory, Institut Curie, 26 Rue d’Ulm, Paris, France; 3 Centre of Complement and Inflammation Research, Imperial College London, Du Cane Road W12 0NN, London, UK

## Abstract

**Background:**

The oxidative burst is one of the major antimicrobial mechanisms adopted by macrophages. The WKY rat strain is uniquely susceptible to experimentally induced macrophage-dependent crescentic glomerulonephritis (Crgn). We previously identified the AP-1 transcription factor JunD as a determinant of macrophage activation in WKY bone marrow-derived macrophages (BMDMs). JunD is over-expressed in WKY BMDMs and its silencing reduces Fc receptor-mediated oxidative burst in these cells.

**Results:**

Here we combined *Jund* RNA interference with microarray analyses alongside ChIP-sequencing (ChIP-Seq) analyses in WKY BMDMs to investigate JunD-mediated control of macrophage activation in basal and lipopolysaccharide (LPS) stimulated cells. Microarray analysis following *Jund* silencing showed that *Jund* activates and represses gene expression with marked differential expression (>3 fold) for genes linked with oxidative stress and IL-1β expression. These results were complemented by comparing whole genome expression in WKY BMDMs with *Jund* congenic strain (WKY.L*Crgn2*) BMDMs which express lower levels of JunD. ChIP-Seq analyses demonstrated that the increased expression of JunD resulted in an increased number of binding events in WKY BMDMs compared to WKY.L*Crgn2* BMDMs. Combined ChIP-Seq and microarray analysis revealed a set of primary JunD-targets through which JunD exerts its effect on oxidative stress and IL-1β synthesis in basal and LPS-stimulated macrophages.

**Conclusions:**

These findings demonstrate how genetically determined levels of a transcription factor affect its binding sites in primary cells and identify JunD as a key regulator of oxidative stress and IL-1β synthesis in primary macrophages, which may play a role in susceptibility to Crgn.

## Background

Macrophages are efficient phagocytes of the immune system that produce reactive oxygen species (ROS) during the phagocytosis of pathogens, considered as a marker of cell activation. The well-established classical pathway of macrophage activation induced by interferon (IFN)-γ and/or lipopolysaccharide (LPS) is known to play a vital role in host defence during inflammation. Macrophages activated in this manner express high levels of proinflammatory cytokines and reactive oxygen and nitrogen intermediates that are crucial in the defence against intracellular pathogens [1,2]. The AP-1 transcription factor plays a key role in regulating cell growth and environmental stress responses [3-5]. In classically activated (M1) macrophages, AP-1 plays a central role together with NF-κB in signal-dependant gene expression that is crucial for innate immunity [6]. JunD is a member of AP-1 that is constitutively expressed and has been previously shown to protect cells from oxidative stress and to reduce tumour angiogenesis by limiting the production of ROS [7]. The chronic oxidative stress generated by the inactivation of JunD, has been shown to promote aging and increase tumour development [8,9]. In various tissues, including the kidney, the absence of JunD led to the over-expression of hypoxia inducible factor (HIF)-target genes in podocytes, most likely as a result of increased oxidative stress [10].

Wistar Kyoto (WKY) rats are uniquely susceptible to nephrotoxic nephritis (NTN), a rat model of crescentic glomerulonephritis (Crgn) [11]. The macrophages of this strain show a 20-fold increase in *Jund* mRNA expression as well as increased specific JunD protein binding to AP-1 consensus sequence nucleotides (5^′^-TGAGTCA-3^′^) when compared with the NTN-resistant LEW strain [12]. In addition WKY BMDMs show greater superoxide anion production when stimulated with PMA (unpublished observations) and significantly increased NOS2 expression [13] when stimulated with LPS, suggesting that the macrophages of this strain have a genetically determined pro-inflammatory phenotype characterised by increased oxidative stress. We have previously shown that JunD is a determinant of the macrophage oxidative burst associated with crescentic glomerulonephritis. In a genome-wide linkage analysis and haplotype analysis for NTN-related phenotypes in WKY and LEW rats, we delineated a minimal genomic region of 130 kb on rat chromosome 16 where *Jund* was the only markedly over-expressed transcript. The functional role of JunD was established by siRNA knock-down of *Jund* in WKY BMDMs [12] which resulted in reduced Fc receptor mediated oxidative burst confirming the previously reported antioxidant role of JunD in other tissues [7,9]. Furthermore, the role of JunD in TLR4-induced primary human macrophage activation was established. siRNA knockdown of *JUND* in these cells resulted in a significantly reduced secretion of TNFα, IL-6 and IL-10 [12]. One possible mechanism for this was suggested by Smolinska and colleagues who showed that Hck kinase mediates TLR4-induced transcription of both TNF and IL-6 through binding of AP-1 heterodimers composed of c-Fos and JunD [14]. Based on these results, we hypothesised that JunD controls respiratory burst and the related oxidative stress in basal and classically activated (LPS/TLR4, M1) macrophages.

To identify genes and pathways regulated by JunD-mediated macrophage activation in WKY BMDMs, we have carried out microarray-based gene expression studies following siRNA knock down of *Jund* in basal and LPS-stimulated conditions. ChIP-Seq analysis was performed on basal and LPS-stimulated WKY BMDMs and used to complement the microarray results in order to identify primary JunD targets. ChIP-Seq and microarray analyses were also carried out in a *Jund* congenic strain (WKY.L*Crgn2*) known to have reduced JunD mRNA and protein levels [12]. In this strain, the *Jund* locus was transferred from the Lewis strain into the WKY strain by back-crossing over nine generations. Genome-wide integration of all datasets identified primary JunD-target genes and a regulatory network involved in oxidative stress and IL-1β expression in macrophages leading to increases in mature IL-1β production in BMDMs and glomeruli from the WKY strain.

## Results

### *Jund* regulates macrophage gene expression that controls primarily oxidative stress and IL-1β synthesis

**Figure 1:**
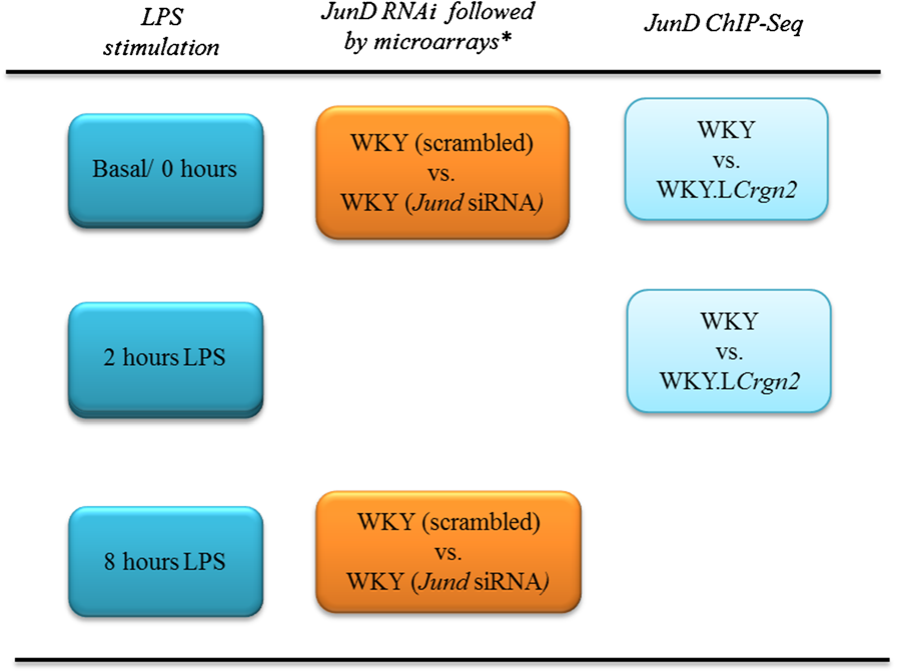
**Strategy employed to identify primary JunD targets in bone marrow derived macrophages.** We performed *Jund* RNAi and compared whole genome expression profiling in macrophages (basal and stimulated with LPS, 100ng/ml, 8h) transfected with scrambled siRNA to those transfected with *Jund* siRNA. ChIP-Seq analysis was performed in WKY and WKY.L*Crgn2* BMDMs (basal and stimulated with LPS, 100ng/ml, 2h). Transcripts showing a fold change > 3 in the RNAi dataset were examined for JunD/AP1 peaks.

**Figure 2:**
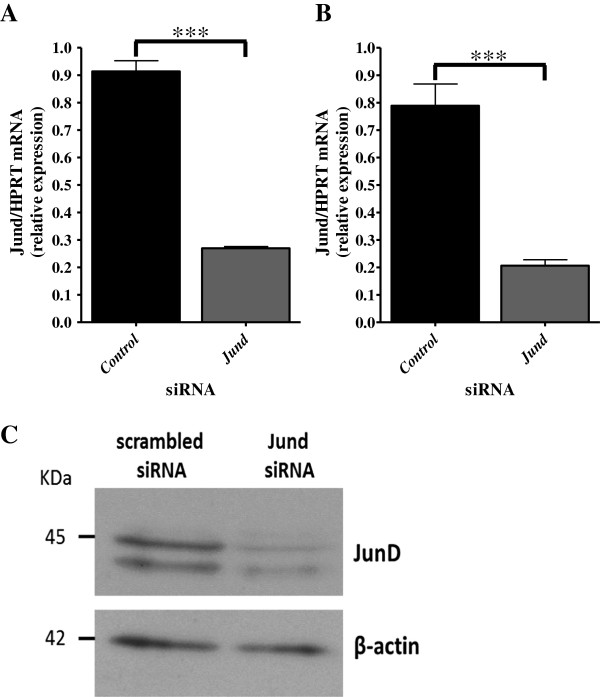
**siRNA mediated knockdown of *Jund*.** Assessment of the efficiency of siRNA knockdown of *Jund* in the unstimulated state (**A**, **C**) and following eight hours LPS stimulation (**B**) using qRT-PCR and Western blotting. siRNA experiments were performed in 4 different WKY rats in triplicate. ***P<0.001 using two tailed unpaired t-test to compare BMDMs transfected for 48 hours with either scrambled control siRNA or *Jund* siRNA. The Western blot (**C**) for JunD is representative of four different *Jund* silencing experiments in WKY BMDMs and is demonstrated alongside β-actin loading control.

**Figure 3:**
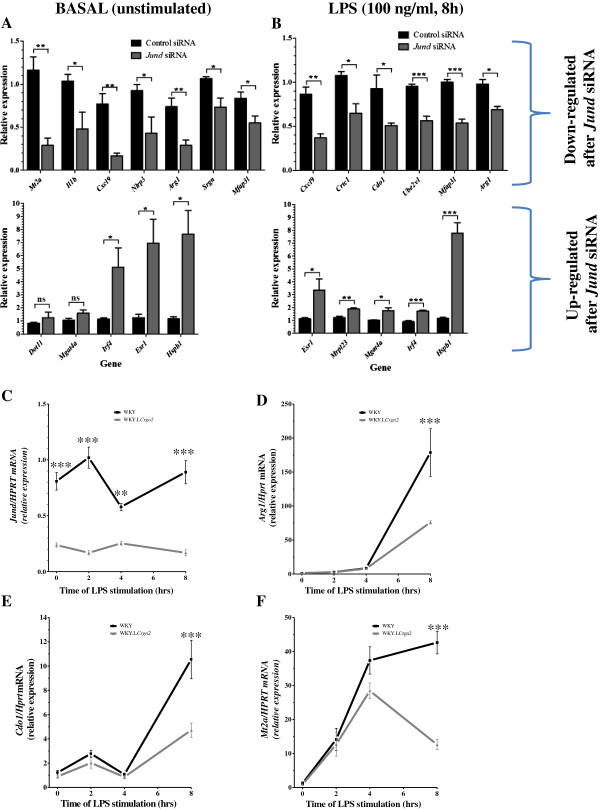
**Validation of microarray results confirms that JunD has both activatory and repressive roles in controlling gene expression linked with oxidative stress.** Validation of differentially expressed genes were carried out using four biological replicates with three technical amplification replicates per siRNA type for the unstimulated (**A**) and eight hour LPS stimulated (**B**) data sets. Relative gene expression was normalised to *Hprt* and used to generate fold change values. *P<0.05; **P<0.01;***P<0.001 using a two-tailed unpaired t-test to detect statistically significant differences between the siRNA groups. Error bars represent standard error of the mean. Confirmation of the differential expression of *Jund* between WKY and WKY.L*Crgn2* BMDMs (**C**) and key JunD targets; *Arg1* (**D**), *Cdo1* (**E**) and *Mt2a* (**F**), influenced by Jund siRNA knockdown with >2 fold change in expression that were also differentially expressed between WKY and WKY.L*Crgn2* BMDMs over a timecourse of LPS stimulation. Samples from the WKY and WKY.L*Crgn2* strains were amplified using a set of four biological replicates with three technical replicates per sample. ***P<0.001 statistically significantly different to WKY using a two way ANOVA to compare the overall timecourse with Bonferonni’s post-tests to compare individual time points.

A description of the macrophage function related to different levels of JunD expression in different inbred rat is summarised in Table 1. In order to identify genes under the transcriptional control of JunD in primary macrophages, expression levels of *Jund* were first silenced by RNA interference in the WKY BMDMs that over-express JunD (Figure 1) [12]. Following confirmation of knockdown by qRT-PCR and Western blot (Figure 2A and C), the samples were subjected to microarray analysis (Additional file 1: Figure S1A). Because AP1/JunD regulates lipopolysaccharide (LPS)-TLR4 mediated cytokine secretion in primary human macrophages [12,14], microarray analysis was also performed in samples treated with *Jund* siRNA (48h) and stimulated with LPS for 8 h (Figure 2B and Additional file 1: Figure S1B). Genome-wide analysis of the BMDM transcriptome identified 1672 differentially expressed genes between unstimulated BMDMs transfected with *Jund* siRNA compared to scrambled control and 1,476 differentially expressed genes following 8 hours of LPS (100ng/ml) stimulation (FDR< 5%). JunD acted both as an activator and as a repressor of gene expression in BMDMs. Transfection of WKY BMDMs with *Jund* siRNA, thereby lowering *Jund* expression, resulted in the reduced expression of 868 genes out of 1672 (~ 50%) demonstrating that *Jund* had an activatory role in gene transcription. Alongside this, *Jund* knockdown also increased the expression of 804 genes (~ 50%) demonstrating that *Jund* could also have a repressive effect on transcription. After eight hours of LPS stimulation, 638 genes had reduced expression in the *Jund* siRNA knockdown group compared to controls and 838 genes demonstrated higher expression following *Jund* knockdown. Validation by qRT-PCR of a set of differentially expressed genes following *Jund* siRNA knock-down that encompassed a range of fold change differences between the two siRNA groups confirmed the microarray findings (Figure 3A and B, Additional file 2: Table S1) in 21 out of the 23 (91%) genes selected. The directional change (activation or repression) of the microarray data was confirmed in all 23 genes.

**Table 1:** JunD levels and macrophage oxidative burst in rat strains used for the combined ChIP-Seq and transcriptome approach

**Strain**	*JunD *levels in macrophages^*^	Macrophage activation ^**^
WKY	**+++**	**+++**
WKY.L*Crgn2*	**+**	**+**
Lewis	**+**	**+**

* *Jund* expression levels and JunD/AP-1 protein binding measured by qRT-PCR and TransAm assay [12].

** Macrophage activation assessed by Fc receptor mediated oxidative burst [12].

Amongst the genes showing the most marked expression changes following *Jund* siRNA knock-down in WKY BMDMs, 11 genes were found to have greater than threefold differences in expression between the two siRNA groups (scrambled vs. *Jund* siRNA) in the unstimulated state and 2 genes were identified in the LPS stimulated state (Table 2). Interestingly, the individual functions of the majority of these genes belong to two main categories: oxidative stress (*Mt2a*[15], *Lcn2*[16], *Vcan*[17], *Hspb1*[18], *Prkca*[19]) and IL-1β synthesis (*Klrb1a*[20], *Il1b*, *Nlrp3*[21]). This suggests that JunD has a primary role in regulating macrophage gene expression associated with oxidative stress and IL-1β synthesis. To investigate this further, we performed genome-wide expression analysis by microarrays over an eight hour time course of LPS stimulation in WKY and WKY.L*Crgn2* BMDMs (Additional file 1: Figure S1C). The latter is a *Jund* congenic strain generated by introgression of the *Jund* locus on chromosome 16 from the Lewis donor onto the WKY recipient genome. WKY.L*Crgn2* rats have significantly less *Jund* mRNA and JunD protein levels in their BMDMs when compared with parental WKY BMDMs (Table 1). The microarray results between the WKY and WKY.L*Crgn2* BMDMs identified that 830 genes were differentially expressed over the eight hour timecourse. They were functionally associated with multiple immune terms focused on responses to stimuli including LPS and the regulation of cell activation (Table 3) and were validated by qRT-PCR (Additional file 1: Figure S2). A set of 201 genes that were common with the *Jund* siRNA knockdown was identified. Amongst these, seven transcripts showed a fold change greater than 2 (FDR < 5%) including metallothionein 2A (*Mt2a*), arginase (*Arg1*) and cysteine dioxygenase, type 1 (*Cdo1*), genes associated with oxidative stress. The significant differential expression of these transcripts together with *Jund* between WKY and WKY.L*Crgn2* BMDMs was confirmed by qRT-PCR (Figure 3C-F). Taken together the combined RNAi and congenic whole genome differential expression analysis identifies genes primarily regulating oxidative stress and IL-1β synthesis under transcriptional control of JunD.

**Table 2:** **Microarray results of transcripts demonstrating greater than three-fold difference in expression between *Jund *siRNA and scrambled control siRNA transfected WKY BMDMs**

**Gene symbol**	**Gene name**	**Chr.**	**Fold change**	**FDR (%)**	**Gene Function**
*Basal condition*					
*Klrb1a*	killer cell lectin-like receptor subfamily B member 1B	4	9.0	<0.01	IL-1β synthesis [19]
*Mt2A*	metallothionein 2A	19	4.6	0.63	Oxidative stress [15]
*Il1b*	interleukin 1 beta	3	4.1	2.85	IL-1β synthesis
*Cxcl9*	chemokine (C-X-C motif) ligand 9	14	3.5	<0.01	Inflammation
*Lcn2*	lipocalin 2	3	3.3	<0.01	Oxidative stress [16]
*Vcan*	versican	2	3.3	<0.01	Oxidative stress [17]
*Nlrp3*	NLR family, pyrin domain containing 3	10	3.2	3.01	IL-1β synthesis [21]
*D3ZIY9_RAT*	uncharacterised protein	10	3.1	1.09	-
*Dot1l*	DOT1-like, histone H3 methyltransferase (S. cerevisiae)	7	-3.7	2.30	Inflammation
*Hspb1*	heat shock protein 1	12	-3.8	<0.01	Oxidative stress [18]
*Prkca*	protein kinase C, alpha	10	-4.1	1.83	Oxidative stress [19]
*LPS stimulated condition*					
*Klrb1a*	killer cell lectin-like receptor subfamily B member 1B	4	4.6	<0.01	IL-1β synthesis [20]
*Hspb1*	heat shock protein 1	12	-4.0	<0.01	Oxidative stress [18]

Differentially expressed transcripts with greater than three-fold difference in expression (four technical replicates per strain) were identified at a <5% FDR threshold following 40,000 permutations. Fold changes are of control siRNA versus *Jund* siRNA expression. A positive fold change indicates higher expression in BMDMs transfected with scrambled control siRNA i.e. with a higher level of *Jund* expression compared to *Jund* siRNA. Abbreviations: Chr.; chromosome, FDR: false discovery rate.

**Table 3:** **Gene ontology analysis for genes demonstrating differential expression over the eight hour LPS timecourse between WKY and WKY.L*Crgn2 *BMDMs**

**Gene ontology term (BP_FAT or KEGG pathway)**	**Genes (n)**	**Fold Enrichment**	**Bonferroni corrected P-Value**
GO:0010033~response to organic substance	84	2.23	9.00E-09
GO:0009725~response to hormone stimulus	53	2.57	1.59E-06
GO:0009611~response to wounding	46	2.68	6.71E-06
GO:0009719~response to endogenous stimulus	53	2.28	9.90E-05
GO:0001775~cell activation	31	3.18	1.08E-04
GO:0043434~response to peptide hormone stimulus	29	3.24	2.22E-04
GO:0050865~regulation of cell activation	25	3.63	2.44E-04
GO:0002237~response to molecule of bacterial origin	21	4.02	6.34E-04
GO:0002694~regulation of leukocyte activation	23	3.49	0.0018
GO:0032496~response to lipopolysaccharide	19	3.95	0.0036
GO:0045767~regulation of anti-apoptosis	11	7.37	0.0039
GO:0045321~leukocyte activation	26	3.04	0.0039
GO:0001817~regulation of cytokine production	22	3.42	0.0047
GO:0009617~response to bacterium	26	3.00	0.0052
GO:0006954~inflammatory response	26	2.98	0.0056
GO:0048545~response to steroid hormone stimulus	31	2.65	0.0058
GO:0043067~regulation of programmed cell death	54	1.96	0.0079
GO:0010941~regulation of cell death	54	1.96	0.0085
GO:0031667~response to nutrient levels	30	2.64	0.0095
GO:0042981~regulation of apoptosis	53	1.95	0.012
GO:0009991~response to extracellular stimulus	31	2.54	0.014
GO:0002684~positive regulation of immune system process	26	2.80	0.018
GO:0051249~regulation of lymphocyte activation	20	3.37	0.020
GO:0007568~aging	20	3.35	0.022
GO:0006952~defense response	36	2.28	0.024
GO:0045768~positive regulation of anti-apoptosis	9	8.03	0.027
GO:0007565~female pregnancy	16	3.94	0.033
GO:0014070~response to organic cyclic substance	24	2.85	0.033
GO:0008283~cell proliferation	27	2.62	0.036
GO:0046649~lymphocyte activation	21	3.09	0.041
GO:0042592~homeostatic process	53	1.86	0.044

Enrichment for biological process functional annotation terms and KEGG canonical pathways used a Bonferroni corrected p-value threshold of <0.05. Abbreviations: BP_FAT, subset of Biological Process Gene Ontology (GO) terms generated by DAVID; n, number of involved genes.

### JunD expression levels determine the extent of the JunD cistromes

**Figure 4:**
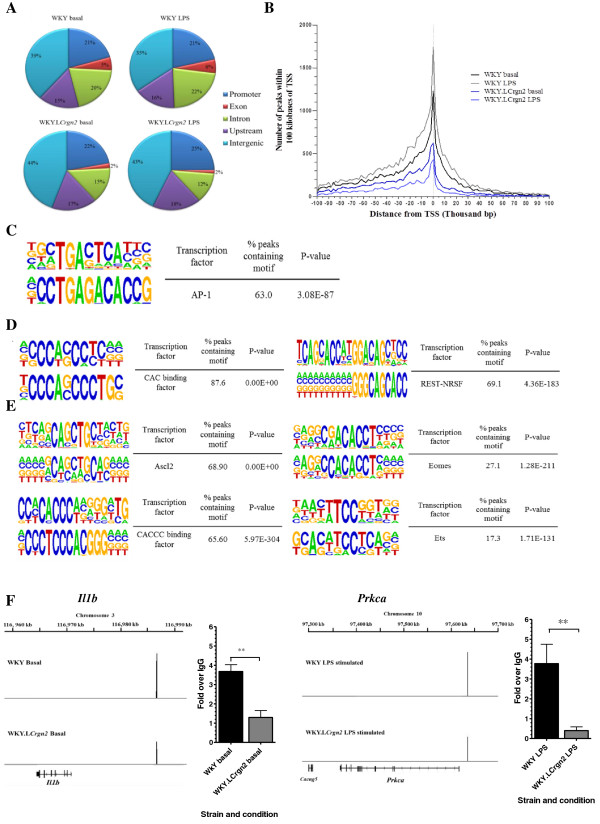
**Genetically determined differences in Jund expression alter the JunD cistrome and identifies primary JunD targets.** (**A**) Distribution of JunD-peaks relative to transcriptional start sites (TSS) of Ensembl genes. Promoter region defined as 20 kilobases (kb) upstream of the TSS, upstream region between 10kb and 50kb upstream from TSS. (**B**) Occurrence of peaks within 100 kilobases of the TSS. (**C**) Twelve base pair AP-1 motif identified by *de novo* motif analysis present in 63% peaks. *De novo* motif analysis using HOMER identified two *de novo* motifs in basal WKY BMDMs (**D**) and four motifs in LPS stimulated WKY BMDMs (**E**). The *de novo* motif identified is displayed on the bottom of each the pair of motifs, the matched consensus motif for a transcription factor on the top. (**F**) *Il1b* and *Prkca* confirmed as primary JunD targets by qPCR validation. The aligned reads comprising peak passing the posterior probability threshold of 0.9 for each JunD-bound gene in the WKY strain in the LPS stimulated state for *l1b* and the basal state for *Prkca* are shown in genome browser views along with the peak in the WKY.L*Crgn2* strain. Samples from WKY and WKY.L*Crgn2* strains were amplified using three biological replicates with three technical replicates per sample. Results expressed as mean fold change over IgG. *P<0.05; **P<0.01; using a one-tailed unpaired t-test to detect statistically significant differences between the strain and condition pairs. Error bars represent standard error of the mean.

We next combined our microarray approaches investigating JunD-mediated transcriptional control of genes with a cistrome analysis of JunD between basal and two hour LPS stimulated WKY and WKY.L*Crgn2* BMDMs using ChIP-Seq (Figure 1). The aligned sequencing reads (Additional file 2: Table S2) were analysed using BayesPeak [22,23] in order to identify areas of sequencing enrichment that signified JunD-binding events, termed peaks. This analysis identified a greater number of peaks in WKY basal and LPS-stimulated BMDMs compared to WKY.L*Crgn2* BMDMs in both conditions (Table 4). Peaks were linked to a gene if they were located within 20 kilobases of the transcriptional start site or were located within the gene body. This meant that genetically determined differences in JunD levels resulted in an almost 50% reduction in the number of ChIP-Seq peaks that were linked to a protein coding gene in the basal state and an 87% reduction after LPS stimulation (Table 4). Fourteen peaks were successfully validated by ChIP-qPCR in WKY BMDMs with all the peaks showing at least two-fold enrichment for JunD above background (Additional file 1: Figure S3A and S3B). The reduced level of JunD expression in the WKY.L*Crgn2* strain was reflected in the lower levels of enrichment for the seven peaks analysed (Additional file 1: Figure S3C). The majority of ChIP-Seq peaks in all the strains were located in intergenic regions (35-44% across the datasets) whilst the WKY BMDMs had a higher proportion of JunD peaks within the body of the gene (exon or intron) compared to WKY.L*Crgn2* BMDMs in both the unstimulated and LPS stimulated states (Figure 4A). The distance from the nearest gene to each peak was calculated and peaks were found to be preferentially located close to the TSS in both strains (Figure 4B). *De novo* motif discovery using HOMER identified a 12 base pair motif in 63% of peaks in WKY LPS stimulated BMDMs with a strong similarity to the consensus AP-1 motif that has been previously recognised and shown to be functionally active [24] (Figure 4C). Additional motifs identified included CAC binding and REST/NRSF in basal WKY BMDMs (Figure 4D) and the Ascl2, CACCC binding motif for KLF factors, Eomes and Ets (Figure 4E) in LPS stimulated WKY BMDMs. Furthermore the JunD binding to the promoters of *Il1b* and *Prkca*, two of the most markedly differentially expressed transcripts following *Jund* siRNA were confirmed by ChIP-qPCR in both the basal and LPS stimulated conditions (Figure 4F, Additional file 1: Figure S4)

**Table 4:** **JunD binding events in WKY and WKY.L*Crgn2 *BMDMs**

**Dataset**	**Total number of peaks identified**	**Number of peaks linked to a gene**	**Number of genes containing at least one JunD peak**
WKY basal	27124	12522	5339
WKY LPS	36687	18124	7612
WKY.L*Crgn2* basal	16593	6408	2606
WKY.L*Crgn2* LPS	8689	3361	1022

Peaks were identified using BayesPeak v1.1.3 [22,23] using a posterior probability threshold of 0.9. Peaks were linked to a gene if they were located within 20 kilobases of the transcriptional start site or were located within the body of the gene.

Gene ontology analysis of the JunD-bound genes in the different strains and states identified marked similarities between the enriched terms in basal WKY BMDMs (Additional file 2: Table S3) and WKY.L*Crgn2* BMDMs (Additional file 2: Table S4) as well as LPS stimulated WKY.L*Crgn2* BMDMs (Additional file 2: Table S5) covering multiple autoimmune disease pathways and core macrophage functions such as antigen processing. This suggested that regardless of expression level, JunD binds to genes involved in core macrophage processes. Following LPS stimulation in WKY BMDMs enrichment was seen for immune processes linked with the response of the macrophage to stimulation such as intracellular signalling cascades and the MAPK signalling pathway (Additional file 2: Table S6) highlighting the role played by JunD in the LPS response.

### Integration of microarray and ChIP-Seq datasets identifies primary JunD targets in macrophages

**Figure 5:**
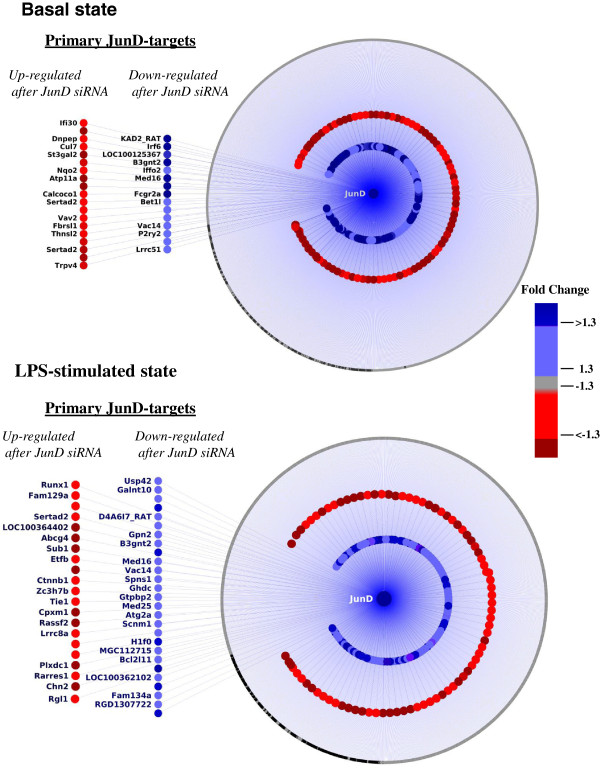
**Integrative analysis identifies primary JunD targets in basal and LPS stimulated BMDMs.***Jund* gene expression patterns in WKY and WKY.L*Crgn2* BMDMs over the LPS time course were used for Spearman correlation analysis with the rest of the transcripts on the microarrays (P < 0.001 cut off). The correlated genes were used for building the JunD target gene networks and selected based on i. significant differential expression following *Jund* siRNA knockdown (both basal and after LPS stimulation FDR< 5%), ii. presence of the JunD ChIP-Seq peak in WKY BMDMs (both basal and after LPS stimulation). The outer ring represents all the transcripts correlating with *Jund* expression levels (P<0.001) in WKY and WKY.L*Crgn2* BMDMs (1445 transcripts, 75%, indicated in grey circles) over the LPS time course. Transcripts associated with a JunD ChIP-Seq peak (within 20kb of TSS or within the gene body) are shown as black circles (232 transcripts, 12%). The transcripts correlating with *Jund* expression levels and down-regulated following *Jund* siRNA (basal, upper panel) are shown in blue (125 transcripts, 6.5%); and those up-regulated (basal, upper panel) are shown as red circles (116 transcripts, 6%). Primary JunD targets in basal BMDMs are given with the gene names and show a JunD ChIP-Seq peak (24 transcripts, basal state, upper panel). This analysis was repeated for the LPS-stimulated macrophages taking into account JunD siRNA and ChIP-Seq datasets in LPS stimulated macrophages and identified 36 primary Jund targets (lower panel).

In order to identify the set of primary JunD targets, we integrated all the genomic datasets and identified the genes that correlated closely with the expression pattern of *Jund* during the LPS timecourse that were also differentially expressed following siRNA knockdown of *Jund* and showed a ChIP-Seq peak in WKY BMDMs. This identified two major networks (basal and LPS) with 24 genes in the basal state (Figure 5, upper panel) and 36 genes after LPS stimulation (Figure 5, lower panel) as primary JunD targets through which JunD mediates its effect on macrophage activation. The primary JunD targets correlate with *Jund* expression levels in two independent datasets (microarray analysis on WKY and WKY.L*Crgn2* BMDM LPS time course and microarray on *Jund* siRNA in WKY BMDMs) and have a JunD ChIP-Seq peak in WKY BMDMs. These are genes where the expression is under direct control of JunD/AP1 binding, correlating with cellular JunD levels. The transcription factor Runx1 was identified as primary Jund targets together with several genes involved in oxidative stress such as *Trpv4, Vav2*, *Ifi30*, *Nqo2,* and *P2ry2, Ctnnb1* and *Bcl2l1* Additional file 1: Figure S5).

### JunD expression levels determine active IL-1β secretion in primary macrophages and nephritic glomeruli

**Figure 6:**
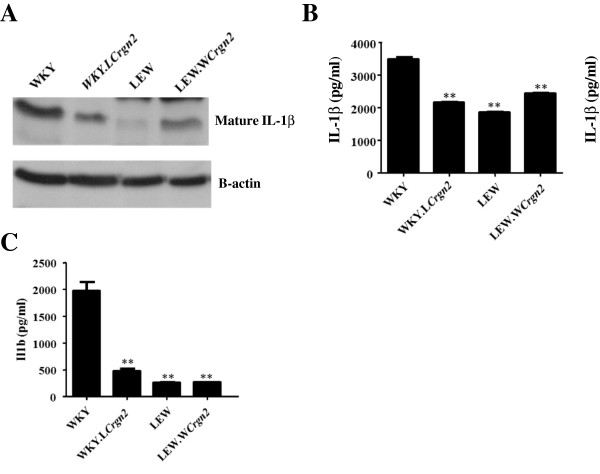
**JunD expression levels control the production of mature IL-1β in BMDMs and nephritic glomeruli.** (**A**) Western blot of mature IL-1β expression in WKY, WKY.L*Crgn2*, LEW and LEW.W*Crgn2* BMDMs primed with LPS and stimulated with ATP. The result of 3 independent experiments is demonstrated alongside β-actin loading control. ELISAs for IL-1β in LPS primed and ATP activated BMDMs (**B**) and in nephritic glomeruli (**C**). **P<0.01 statistically significantly different to WKY using a one way-ANOVA with Bonferonni’s post-tests.

Since the previous role of JunD is regulating macrophage oxidative burst is known [12] and our data shows that JunD regulates genes involved in IL-1β synthesis (*Il1b*, *Nlrp3*, *Klrb1a*), we investigated whether JunD regulates active IL-1β secretion following Nlrp3-inflammasome activation in primary rat macrophages. Western Blot analysis of mature IL-1β in BMDMs primed with LPS and activated by ATP demonstrated a reduction in mature IL-1β between WKY and WKY.L*Crgn2* BMDMs (Figure 6A). Importantly, there was a marked increase in mature IL-1β production in LEW.W*Crgn2* BMDMs compared to LEW confirming that the *Jund* congenic interval was able to alter mature IL-1β production (Figure 6A). These results were confirmed by ELISA for IL-1β in LPS primed and ATP stimulated BMDMs which showed significant differences between the production of IL-1β by WKY BMDMs compared to WKY.L*Crgn2* and LEW BMDMs (Figure 6B). The production of IL-1β in nephritic glomeruli from WKY, WKY.L*Crgn2*, LEW and LEW.W*Crgn2* rats was examined (Figure 6C). This showed a significant reduction in IL-1β production in all the other strains compared to WKY demonstrating a role for the *Crgn2* congenic interval in IL-1β production.

## Discussion

The aim of the present study was to investigate genes regulated by JunD mediating macrophage oxidative burst and pro-inflammatory cytokine production leading to enhanced cell activation in the WKY rat. We used combined microarray and JunD/AP1 ChIP-Seq analyses in primary BMDMs from WKY (NTN-susceptible, high JunD levels, enhanced macrophage oxidative burst) and congenic WKY.L*Crgn2* (reduced NTN, reduced JunD levels, reduced macrophage oxidative burst) rats. Microarray analysis was performed in two experimental settings: following *Jund* siRNA knockdown using lipid-based transfection in WKY BMDMs and between WKY and WKY.L*Crgn2* primary macrophages. ChIP-Seq analysis was also performed between WKY and WKY.L*Crgn2* BMDMs in order to identify primary JunD targets. Microarray and ChIP-Seq experiments were also performed in macrophages activated with LPS to assess the role of JunD in macrophages activated through LPS/TLR4.

JunD reduces tumour angiogenesis by limiting Ras-mediated production of reactive oxygen species (ROS) implicated in the pathophysiology of various diseases, including cancer , regulates genes involved in antioxidant defence and enhances the transcription of VEGF-A, a potent proangiogenic factor [7,9]. In addition JunD deficient mice display persistent hyperinsulinaemia resulting from enhanced pancreatic islet vascularization owing to chronic oxidative stress [9]. In crescentic glomerulonephritis JunD deficiency may cause increased oxidative stress in the glomerular podocytes, leading to altered VEGFA expression and subsequent glomerular injury [10]. In the rat NTN model of Crgn, reduced JunD expression in the congenic WKY.L*Crgn2* strain is associated with 11% reduction in glomerular crescent formation [12,13]. We have carried out a combined ChIP-Seq and transcriptome approach in macrophages, the main effector cells of Crgn, in order to identify JunD targets that may explain its modulatory role. Alongside a general effect by JunD to alter the immune response to LPS in the overall gene sets, the functions of strongly dysregulated (>3 fold) expression changes suggested that primary gene targets of JunD are key effectors in mediating protection from oxidative stress and IL-1β synthesis. Interestingly, this approach identified genes regulating oxidative stress that were previously identified to be under the regulation of JunD in fibroblasts (i.e. cysteine dioxygenase [7]) suggesting that JunD may have common targets in the oxidative stress pathway in different cell types. JunD regulates IL-1β secretion in rat BMDMs and pro-inflammatory cytokine secretion in human monocyte-derived macrophages [12] suggesting that its role of the regulation of the M1 macrophage activation is conserved across the species. Taken together these observations suggest that JunD regulates oxidative stress various diseases with both common and cell specific targets.

Current understanding of JunD function on a genome wide scale has been limited by studies performed on a candidate gene or promoter basis [24-26]. The Encyclopaedia of DNA elements (ENCODE) project has carried out ChIP-Seq for JunD in human transformed cell lines though not in a macrophage or monocyte cell lines [27-29]. JunD has been categorised as a middle-level transcription factor and such factors regulate information-flow bottlenecks and may be the best therapeutic targets for strongly affecting the flow of information through regulatory circuits [29]. The cistrome of JunD was also found to be highly context and cell type specific [29,30]. We used primary macrophages from two inbred rat strains expressing different amounts of JunD in a comparative ChIP-Seq analysis to identify the genomic regions uniquely bound by JunD. The overall landscape of JunD binding in the BMDMs from WKY and WKY.L*Crgn2* BMDMs was comparable to that of other transcription factors studied in primary macrophages stimulated by LPS [31,32]. We found that after LPS stimulation in WKY BMDMs, there was enrichment for genes involved in multiple immune processes linked with responses to multiple different stimuli. In combination with the gene expression data, these findings suggest that genetically determined up-regulation of JunD expression resulted in enhanced macrophage activation in the WKY strain.

*De novo* motif analysis identified additional transcription factor motifs that were unique to the WKY strain suggesting that the increased levels of JunD expression facilitated new partnerships with other transcription factors that did not occur in WKY.L*Crgn2* BMDMs. Key findings included the CACCC-binding domain which binds Krüppel-like family (KLF) transcription factors and are regulators of signalling following activation of macrophages [33-35] and the Ets-1 motif, a factor controlling the expression of cytokine and chemokine genes in a wide variety of cells [36] in LPS stimulated WKY BMDMS. In basal WKY BMDMS a REST-NRSF motif was identified consistent with the functional findings of neuron development and differentiation in unique core JunD-bound genes in WKY BMDMS and potential roles for JunD in excitoxic neuronal cell death and ischaemic injury [37,38].

The main goal of the study was to identify primary JunD target genes responsible for the macrophage activation seen in Crgn-susceptible WKY rat. The siRNA knockdown experiments identified that genes with the greatest changes in expression were associated with IL-1β synthesis. The transcriptional control of *Il1b* expression by JunD was further confirmed by investigating IL-1β secretion upon inflammasome activation in the WKY and reciprocal *Jund* congenic strain BMDMs and nephritic glomeruli (WKY.L*Crgn2* and LEW.W*Crgn2*). Our integrative analysis identified primary JunD targets through which JunD could primarily regulate macrophage activation. In the basal state multiple targets have links with oxidative stress including *Trpv4*[39], *Vav2*[40-42], *Ifi30*[43], *Nqo2*[44] and *P2ry2*[45]. This was also seen after LPS stimulation with transcripts such as *Ctnnb1*[46] and *Bcl2l11*[47] highlighting the key role of JunD in the regulation of oxidative stress in WKY BMDMs. Moreover, the transcription factor *Runx1* (a target in the LPS stimulation group) has been identified as a primary JunD target suggesting that novel transcription factor interactions in macrophages may underlie some JunD-mediated macrophage activation.

## Conclusions

Taken together our data show that genetically determined differences in physiological levels of JunD affect its genome-wide binding patterns in basal and LPS-stimulated primary macrophages. These results identified transcriptional programs underlying JunD-mediated oxidative stress and IL-1β synthesis in primary macrophages which may play a role in susceptibility to Crgn.

## Methods

### Animals

WKY (WKY/NCrl) and LEW (LEW/Crl) rats were purchased from Charles River (Margate, UK). Single congenic rats were generated by introgressing the *Crgn2* QTL from chromosome 16 from a LEW donor onto a WKY recipient background and vice versa as previously described [12]. All procedures were performed in accordance with the United Kingdom Animals (Scientific Procedures) Act.

### BMDM culture

BMDMs were prepared from the femurs of parental and congenic strains using previously described methods [12]. Femurs from adult (8-10 weeks) rats were isolated and flushed with Hanks buffer (Life Technologies). Total bone marrow derived cells were plated and cultured for 5 days in Dulbecco’s modified Eagle’s medium (Life Technologies) containing 25 mM Hepes (Sigma), 25% L929 conditioned medium, 25% decomplemented fetal bovine serum (Biosera), penicillin (100 U/ml, Invitrogen), streptomycin (100 μg/ml, Invitrogen) and _L_-glutamine (2 mM Invitrogen). The cells were characterised as macrophages by ED-1 staining. Basal macrophages were left unstimulated whilst stimulated cells were stimulated with 100 ng/ml lipopolysaccharide (Sigma). Following stimulation, cells for gene expression analysis were homogenized in TRIzol (Invitrogen) and stored at -80°C.

### siRNA inhibition of *Jund* expression

siRNA knockdown was carried out as previously described [12]. Briefly, on day 5 of culture, WKY BMDMs were replated in six-well plates (1x10^6^ cells per well) in DMEM (Invitrogen) overnight and transfected for 48 hours with siGENOME SMARTpool for *Jund* (100 nM, Dharmacon) or siGENOME non-targeting siRNA pool as the scrambled control siRNA using Dharmafect 1 (1:50, Dharmacon) as a transfection reagent in OPTIMEM medium (Invitrogen). The siRNA sequences used in the siGENOME SMARTpool for *Jund* are listed in Additional file 2: Table S7. siRNA knockdown was confirmed with quantitative PCR (detailed below) and Western blotting (Figure 1).

### RNA extraction and microarray preparation

Total RNA was extracted using the TRIzol method and purified using RNeasy Plus spin columns (Qiagen). 100ng of RNA was amplified, labelled and hybridised to Rat Gene 1.0 ST arrays (Affymetrix, Santa Clara, CA, USA) using the Ambion WT Expression Kit (Life Technologies) as per manufacturer’s instructions. For timecourse expression analysis, four BMDM preparations from four biological replicates were used for each timepoint and condition. For siRNA expression analysis, four BMDM preparations from at least two biological replicates were used for each timepoint and condition. The microarray data is available in MIAME-compliant (minimum information about a microarray experiment) format at the Array Express database (http://www.ebi.ac.uk/arrayexpress) under accession code E-MEXP-3469.

### Microarray data analysis

CEL intensity files were produced using GeneChip Operating Software version 1.4 (Affymetrix) and quality tested using the Affymetrix Expression Console v1.1.2. All 32 files in the timecourse data set and 16 files in the siRNA dataset were suitable for further analysis. Probe-level data was normalised using robust multichip average (RMA) [48,49]. A custom definition file was created using up-to-date probe information [50] and filtered to exclude probes containing the 2,520,602 single nucleotide polymorphisms present between the WKY and LEW genomes (Santosh Atanur, MRC Clinical sciences centre, personal communication). The moderated T test with 40,000 permutations implemented in Statistical Analysis of Microarrays (SAM) version 3.0 was used to identify differentially expressed genes at an FDR threshold of 5% and timecourse analysis was performed using EDGE with 40,000 permutations and a 5% FDR threshold [51]. Hierarchical clustering analysis was performed using MultiExperiment Viewer (MeV) v4.8 [52,53] with the Euclidean distance measure. Gene ontology analysis was carried out using the functional annotation tools within DAVID, the Database for Annotation, Visualisation and Integrated Discovery v6.7 [54,55].

### Quantitative PCR

All qPCRs were performed with an ABI 7900 Sequence Detection System (Applied Biosystems, Warrington, UK). A two-step protocol was used as previously described [56] beginning with cDNA synthesis with iScript select (Bio-Rad) followed by PCR using SYBR Green Jumpstart Taq Ready Mix (Sigma). A total of 10ng of cDNA per sample was used. All samples were amplified using a set of 4 biological replicates with three technical replicates used per sample in the PCR. Sequence detection software (SDS) version (Applied Biosystems) was used to obtain the Ct values. Results were analysed using the comparative Ct method and each sample was normalised to the reference gene *Hprt*, to account for any cDNA loading differences. The primer sequences used for the qRT-PCR validation of microarray data are listed in Additional file 2: Table S8.

### Chromatin immunoprecipitation (ChIP)

BMDMs were left in the basal condition or stimulated for 2 hours with 100ng/ml LPS. Cells were fixed for 10 minutes with 1% formaldehyde and ChIP performed using ChIP-IT Express as per manufacturer’s instructions with some modifications. Sonication was carried out using Covaris S2 (Woburn, Massachusetts, USA) in a volume of 300μl sonication buffer with the following settings; 20% duty cycle, intensity 8, 200 cycles/burst, cycle length 30 s for 28 to 30 cycles dependant on cell count. The ChIP lysate was immunoprecipitated using 2 μg of JunD antibody (Santa Cruz sc74-X) or negative IgG control (sc-2026) overnight. Cross links in the immunoprecipitated chromatin and control input chromatin were reversed by heating the samples at 65°C for 5 hours followed by proteinase K digestion for 1 hour. Samples were purified using Qiagen MinElute columns as per manufacturer’s instructions prior to downstream analysis.

### High throughput sequencing

Single read library preparation and high throughput single read sequencing for 36 cycles was carried out on an Illumina Genome Analyser IIx according to the manufacturer’s protocols (Illumina) with some modifications. Each immunoprecipitated sample for library preparation was the product of five separate technical replicates of immunoprecipitation pooled together and purified using Qiagen MinElute columns and quantified using the hsDNA Qubit assay (Invitrogen, Life Technologies, California, USA). A 1:20 dilution of Illumina adaptors was used at the adaptor ligation step to avoid adaptor dimer formation and a 1:2 primer dilution used to prevent dimerization during the PCR amplification stage. The samples were quantified using the hsDNA Qubit assay (Invitrogen, Life Technologies, California, USA) and the size range analysed using a HS DNA chip on a 2100 Bioanalyser (Agilent Technologies, West Lothian, UK) prior to submission for qPCR analysis and cluster generation and sequencing by the CSC/IC Genome Core facility.

### qPCR of ChIP enriched DNA

Immunoprecipitated DNA fragments were analysed by real-time PCR. Primers used are listed in Additional file 2: Table S9. All samples were amplified using a set of 3 biological replicates with three technical replicates used per sample. After an initial denaturation step of 94°C for 2mins, the samples were cycled 40 times at 94°C for 15s, 60°C for 1 min and 72°C for 1 min with data collection performed during the 72°C elongation step. Sequence detection software (SDS) version 2.3 (Applied Biosystems) was used to obtain the Ct values and each sample was analysed with reference to Ct values for matched control ‘Input’ non-immunoprecipitated chromatin. A standard curve of 1:5 dilutions of Input DNA was used to calculate the % Input level of the transcription factor binding at the investigated locus. The standard curve was constructed from the Input DNA sample for the appropriate strain and condition and analysed within the SDS software v2.3. For each test gene the % Input levels were then determined using %total = 2^ΔCt^ × (% of input sample used) where Δ = Ct (Input) - Ct (sample). Fold change over IgG was expressed using 2^-ΔCt^ where ΔCt= ΔCt_Jund_ – ΔCt_IgG._

### ChIP-Seq data analysis

Sequencing of the ChIP-Seq libraries was carried out on the high throughput Illumina Genome Analyzer II. Initial data processing was performed using Illumina Real Time Analysis (RTA) v1.6.32 software (equivalent to Illumina Consensus Assessment of Sequence and Variation, CASAVA 1.6) with default filter and quality settings. Quality filtered reads were then realigned to the reference rat genome (RGSC3.4) using the Burrows Wheeler Alignment tool v0.5.9 (BWA) [57]. Read ends were trimmed if Phred-scaled base quality scores dropped below 20. Reads that uniquely mapped to the reference genome were used to detect areas of enrichment with BayesPeak v1.1.3 [22,23] using a posterior probability threshold of 0.9. A stringent posterior probability threshold of 0.9 was used to filter all bins passing the threshold to form the final contiguous peak regions to produce a more accurate reflection of true peak calls. An over fitting diagnostic was performed using λ_1_ < 0.7 and score < -2.25 to filter out regions which showed no enrichment but had a high enough background for the algorithm to call peaks in. Peak regions were annotated using the gene intervals annotator (GIN) implemented in CARPET using a gene priority approach to give the associated transcript I.D., the associated gene feature and the distance of the peak to the nearest transcriptional start site [58]. HOMER was used to predict motif occurrence within peaks [32,59] with default settings for a maximum motif length of 12 base pairs. The outputs of the peak calling algorithms were visualised in the Integrative Genomics Viewer [60] using custom WIG files generated from the output data generated by BayesPeak. The latter were generated by extending each mapped read by 200 bp and then by using 10 bp bins the overlapping tag count was generated.

### Integrated data analysis and identification of primary JunD targets

JunD gene expression patterns in WKY and WKY.L*Crgn2* BMDMs over the LPS time course were used for Spearman correlation analysis with the rest of the transcripts on the microarrays (P < 0.001 cut off). In order to integrate the three different datasets, the list of significantly correlated set of transcripts was filtered and annotated according to two criteria: significant differential expression following *Jund* siRNA knockdown (both basal and after LPS stimulation) and the presence of the JunD ChIP-Seq peaks in WKY BMDMs (both basal and after LPS stimulation). Two separate networks (basal and LPS) were built with Cytoscape version 2.8.3 showing primary JunD targets.

### Isolation and culture of rat nephritic glomeruli and detection of IL-1β by Western Blot and ELISA

Glomeruli were isolated as previously described [13]. After 48 hours of incubation at 37°C, nephritic glomeruli supernatants were collected and stored at -20°C for IL-1β sandwich ELISA analysis. For IL-1β detection in BMDMs by Western Blotting, cells were primed with LPS (1 μg/ml, 3hours) and stimulated with ATP (5 mM) for 30 minutes. BMDMs were then scraped and both cells and supernatant were collected, filtered using Amicon ultra centrifugal filters for protein purification and concentration (10 kDa cut-off, Millipore, UK) and the concentrated samples were diluted with 5× sample buffer containing 200 mM Tris-HCl, 6% SDS, 2mM EDTA, 4% 2- Mercaptoethanol, 10% glycerol and boiled for 10 minutes. The samples were then resolved by SDS polyacrylamide gel electrophoresis (PAGE) and transferred to an Immobilon-P Transfer Membrane (Millipore). Rabbit polyclonal anti IL-1β (New England BioLabs, UK) was used to detect the mature IL-1β. To assess secreted IL-1β in BMDMs and nephritic glomeruli, cell supernatants were subjected to sandwich ELISA and secreted IL-1β amounts were determined using a standard curve with rat recombinant IL-1β according to manufacturer’s instructions (R and D Systems).

For the confirmation of siRNA knockdown of *Jund*, WKY BMDMs were plated into six-well plates at a density of 1 × 10^6^ cells per well and treated with *Jund* specific siRNA or a scrambled oligonucleotide for 48 hours before total protein was extracted for Western Blot analysis using the above technique. For JunD detection a specific rabbit polyclonal anti-Jund antibody from Santa Cruz Biotechnology (USA) was used. This blot is representative of 4 different experiments performed with 4 biological replicates.

## Competing interests

The authors declare that they have no competing interests.

## Authors’ contributions

RPH, THC, TJA and JB conceived and designed the study. RPH, ZD and JB carried out the laboratory experiments and LG led the microarray and high throughput sequencing laboratory analyses. PKS and RPH carried out the bioinformatic analysis with contributions from SSA and EP. JB wrote the manuscript with RPH and contributions from TJA, THC and FMG. All authors read and approved of the final manuscript.

## Supplementary Material

Additional file 1: Figure S1 Genome-wide expression analysis in basal and LPS stimulated BMDMs. Genome wide expression analysis by microarrays was performed in BMDMs transfected with rat *Jund* or scrambled control siRNA for the unstimulated condition (A) or following eight hours of LPS stimulation (B) in WKY BMDMs and over an eight hour time course of LPS stimulation in WKY and WKY.L*Crgn2* BMDMs (C). Heat maps of hierarchically clustered significantly differentially expressed genes (<5% FDR threshold) are displayed. All experiments were performed in 4 biological replicates for each strain or siRNA transfected. **Figure S2.** Validation of microarray data between WKY and WKY.L*Crgn2* BMDMs over an eight hour LPS stimulation timecourse. Validation of microarray data by qRT-PCR. Samples were amplified using a set of four biological replicates with three technical replicates per sample. Relative gene expression was measured by qRT-PCR and normalised with *Hprt* for WKY and WKY.L*Crgn2* BMDMs. *P<0.05; **P<0.01;***P<0.001 statistically significantly different to WKY using a two way ANOVA to compare the overall timecourse with Bonferonni’s post-tests to compare individual time points. **Figure S3.** ChIP-Seq peak validations by ChIP-qPCR. ChIP-Seq peaks identified at a posterior probability threshold of 0.9 for basal WKY BMDMs were validated by qPCR (A) and for LPS stimulated WKY BMDMs (B) and WKY.L*Crgn2* BMDMs peaks (C). Samples were amplified using a set of biological triplicates with three technical replicates per sample. Results expressed as mean fold change over IgG. **P<0.01, *P<0.05, ns; non-significant using a paired t-test (one-tailed) to compare whether % input for the JunD ChIP qPCR was significantly different to % input for IgG. **Figure S4.***Il1b* and *Prkca* confirmed as primary JunD targets by qPCR validation. The aligned reads comprising peak passing the posterior probability threshold of 0.9 for each JunD-bound gene in the WKY strain in the basal state for *l1b* (A) and the LPS stimulated state for *Prkca* (B) are shown in genome browser views along with the peak in the WKY.L*Crgn2* strain. Samples from WKY and WKY.L*Crgn2* strains were amplified using three biological replicates with three technical replicates per sample. Results expressed as mean fold change over IgG. *P<0.05; **P<0.01; using a one-tailed unpaired t-test to detect statistically significant differences between the strain and condition pairs. Error bars represent standard error of the mean. **Figure S5.** Integrative analysis identifies the transcription factor *Bcl2l11* as a primary JunD target. *Jund* microarray-determined expression patterns in WKY and WKY.L*Crgn2* BMDMs over an eight hour LPS timecourse using four biological replicates per strain were used for Spearman correlation analysis (A) with the rest of the transcripts on the microarrays. The expression of *Bcl2l11* (B) was significantly correlated to the *Jund* expression pattern (Spearman correlation 0.9, corrected p-value=8.6x10^-5^). Significant differential expression of the gene was seen following siRNA knockdown of *Jund* (C). Fold changes are of control siRNA versus *Jund* siRNA expression. The positive fold change indicates higher expression in BMDMs transfected with scrambled control siRNA i.e. with a higher level of *Jund* expression compared to *Jund* siRNA. Abbreviations: Chr.; chromosome, FDR: false discovery rate. Three JunD binding events were identified at a posterior probability threshold of 0.9 in LPS stimulated WKY BMDMs (D) located in the gene promoter and second intron. Click here for file

Additional file 2: Table S1 Validation of differentially expressed genes identified by siRNA microarray data analysis with quantitative PCR. **Table S2.** Sequencing and mapping statistics for ChIP-Seq in WKY and WKY.L*Crgn2* BMDMs. **Table S3.** Gene ontology analysis of JunD-bound genes in basal WKY BMDMs. **Table S4.** Gene ontology analysis of JunD-bound genes in basal WKY.L*Crgn2* BMDMs. **Table S5.** Gene ontology analysis of JunD-bound genes in LPS stimulated WKY.L*Crgn2* BMDMs. **Table S6.** Gene ontology analysis of JunD-bound genes in LPS stimulated WKY BMDMs. **Table S7.** Sequences of the four individual siRNAs that comprise siGENOME SMARTpool M-092127-00-0010 (Dharmacon). **Table S8.** Primer sequences used for qRT-PCR validation of microarray data. **Table S9.** Primer sequences used for qPCR validation of ChIP-Seq data. Click here for file
