# Supplementary material for: Combined ChIP-Seq and transcriptome analysis identifies AP-1/JunD as a primary regulator of oxidative stress and IL-1β synthesis in macrophages
Source: BMC Genomics. 2013 Feb 11;14:92. doi: 10.1186/1471-2164-14-92 (PMC3608227; doi:10.1186/1471-2164-14-92)
Supplement: Additional file 1: Figure S1 — Genome-wide expression analysis in basal and LPS stimulated BMDMs. Genome wide expression analysis by microarrays was performed in BMDMs transfected with rat Jund or scrambled control siRNA for the unstimulated condition (A) or following eight hours of LPS stimulation (B) in WKY BMDMs and over an eight hour time course of LPS stimulation in WKY and WKY.LCrgn2 BMDMs (C). Heat maps of hierarchically clustered significantly differentially expressed genes (<5% FDR threshold) are displayed. All experiments were performed in 4 biological replicates for each strain or siRNA transfected. Figure S2. Validation of microarray data between WKY and WKY.LCrgn2 BMDMs over an eight hour LPS stimulation timecourse. Validation of microarray data by qRT-PCR. Samples were amplified using a set of four biological replicates with three technical replicates per sample. Relative gene expression was measured by qRT-PCR and normalised with Hprt for WKY and WKY.LCrgn2 BMDMs. *P<0.05; **P<0.01;***P<0.001 statistically significantly different to WKY using a two way ANOVA to compare the overall timecourse with Bonferonni’s post-tests to compare individual time points. Figure S3. ChIP-Seq peak validations by ChIP-qPCR. ChIP-Seq peaks identified at a posterior probability threshold of 0.9 for basal WKY BMDMs were validated by qPCR (A) and for LPS stimulated WKY BMDMs (B) and WKY.LCrgn2 BMDMs peaks (C). Samples were amplified using a set of biological triplicates with three technical replicates per sample. Results expressed as mean fold change over IgG. **P<0.01, *P<0.05, ns; non-significant using a paired t-test (one-tailed) to compare whether % input for the JunD ChIP qPCR was significantly different to % input for IgG. Figure S4.Il1b and Prkca confirmed as primary JunD targets by qPCR validation. The aligned reads comprising peak passing the posterior probability threshold of 0.9 for each JunD-bound gene in the WKY strain in the basal state for l1b (A) and the LPS stimulated state for Prkca (B) [file 1471-2164-14-92-S1.pptx]

## Slide 1
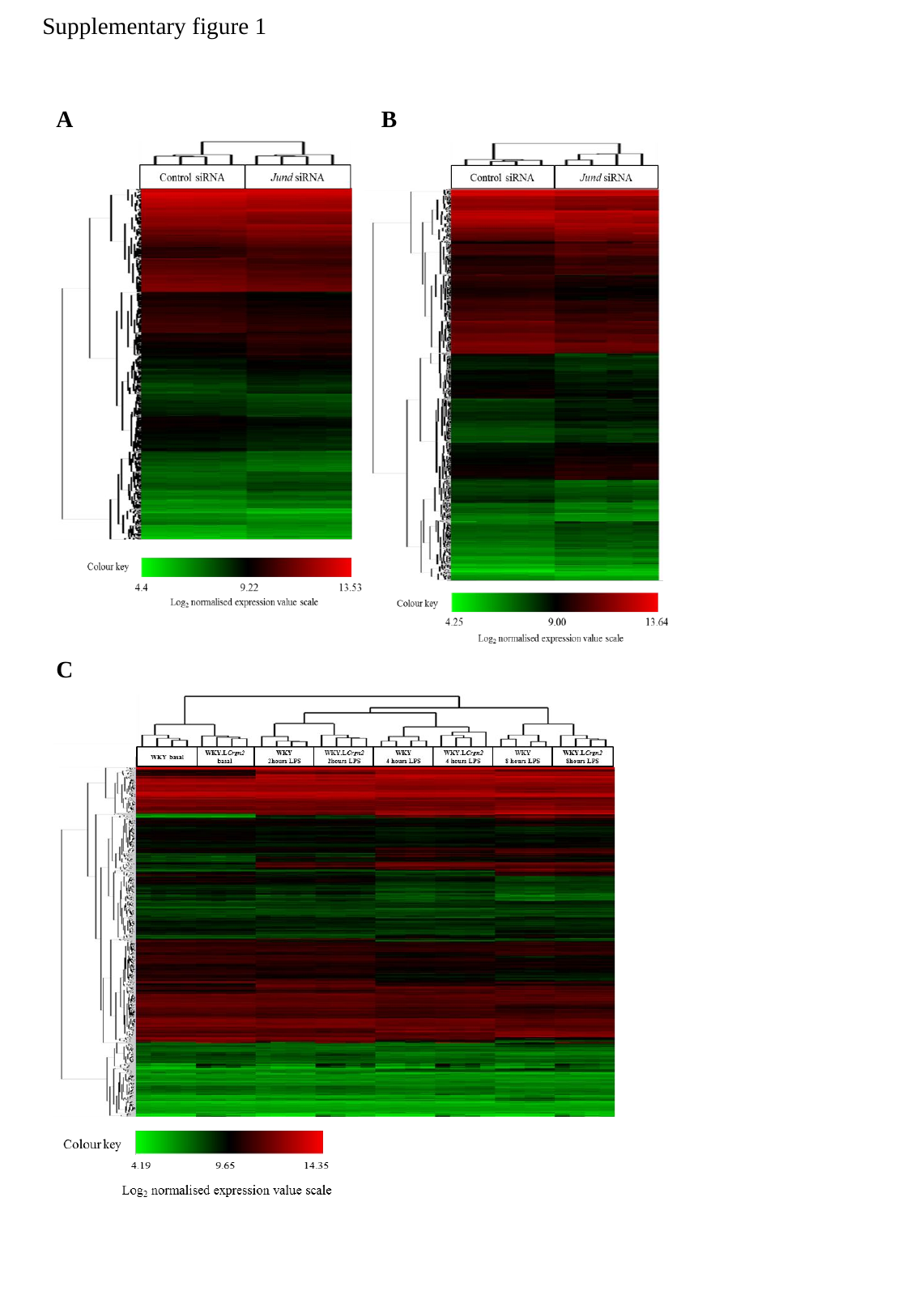

Supplementary figure 1
A
B
C

## Slide 2
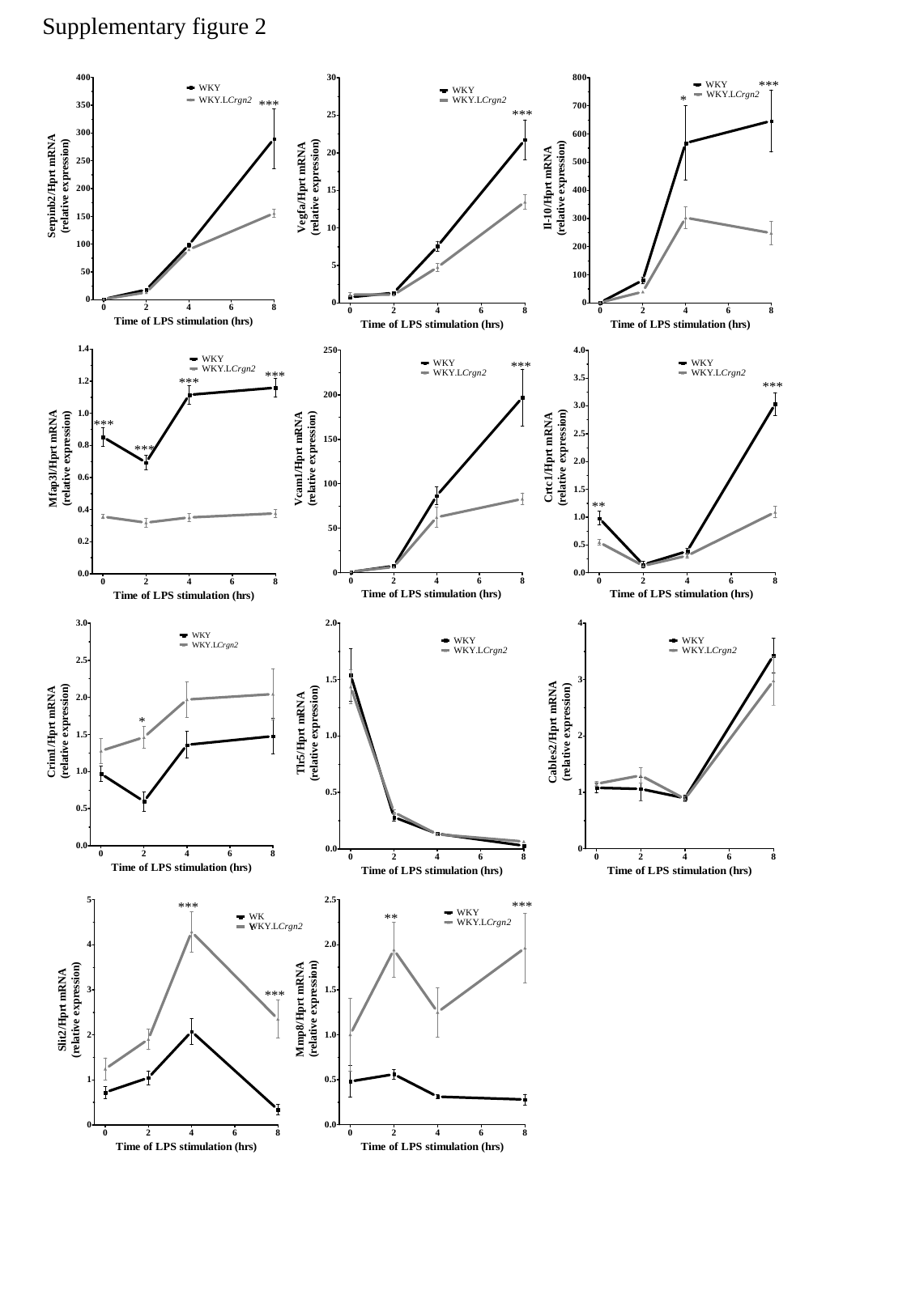

Supplementary figure 2
***
***
***
*
***
***
***
***
***
***
**
*
***
***
**
***

## Slide 3
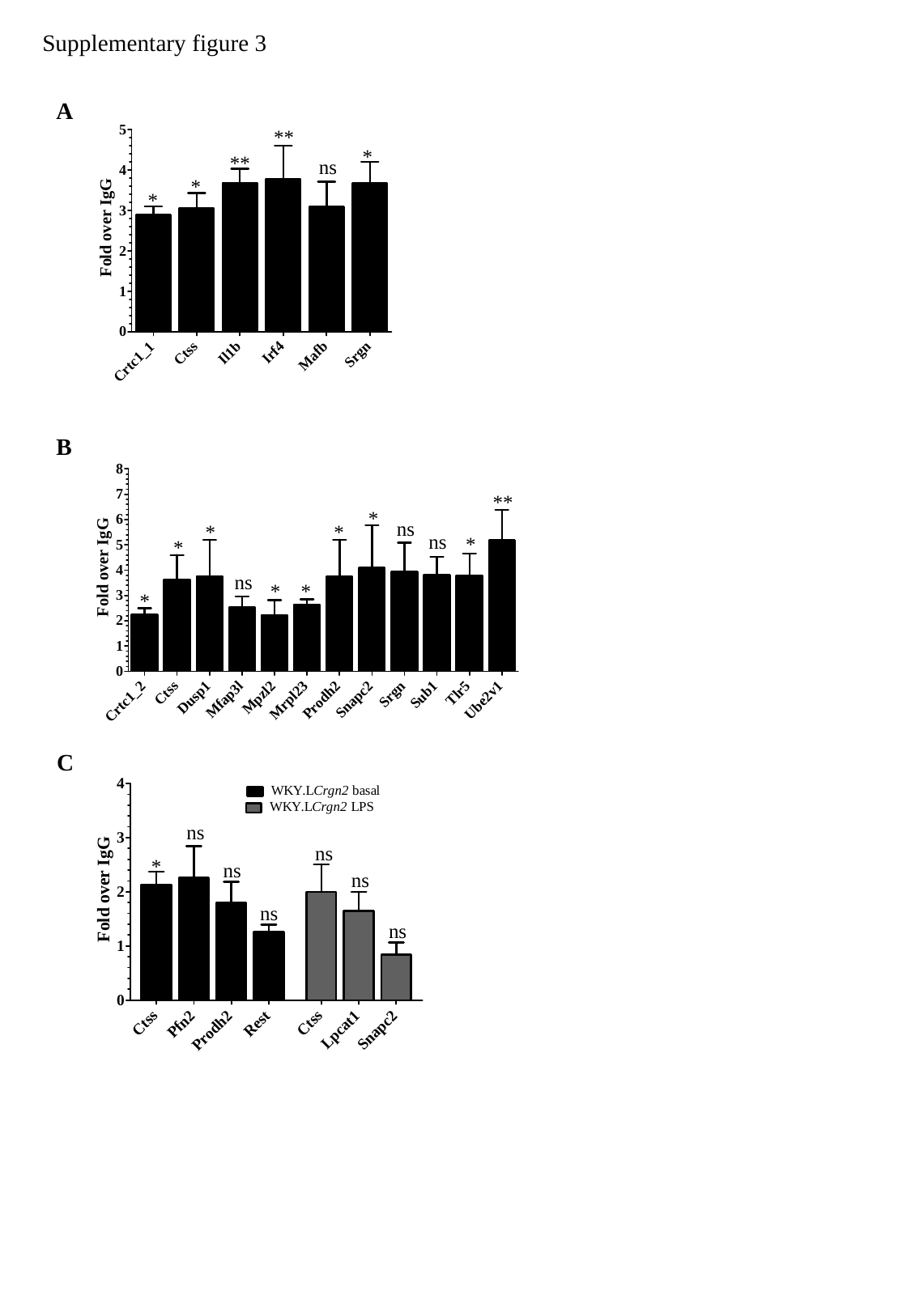

Supplementary figure 3
A
**
*
**
ns
*
*
B
**
*
ns
*
*
ns
*
*
ns
*
*
*
C
ns
ns
*
ns
ns
ns
ns

## Slide 4
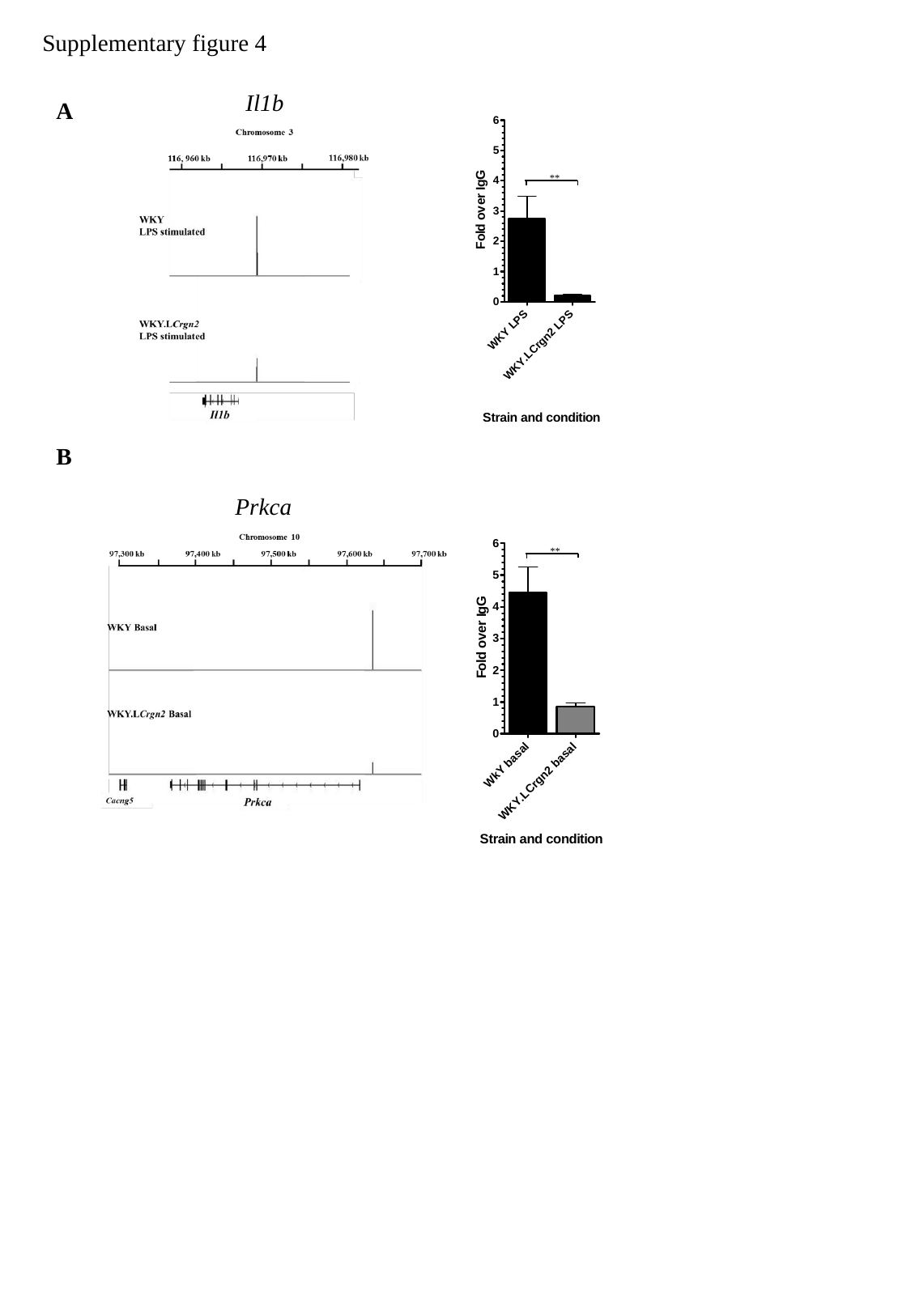

Supplementary figure 4
Il1b
A
**
B
Prkca
**

## Slide 5
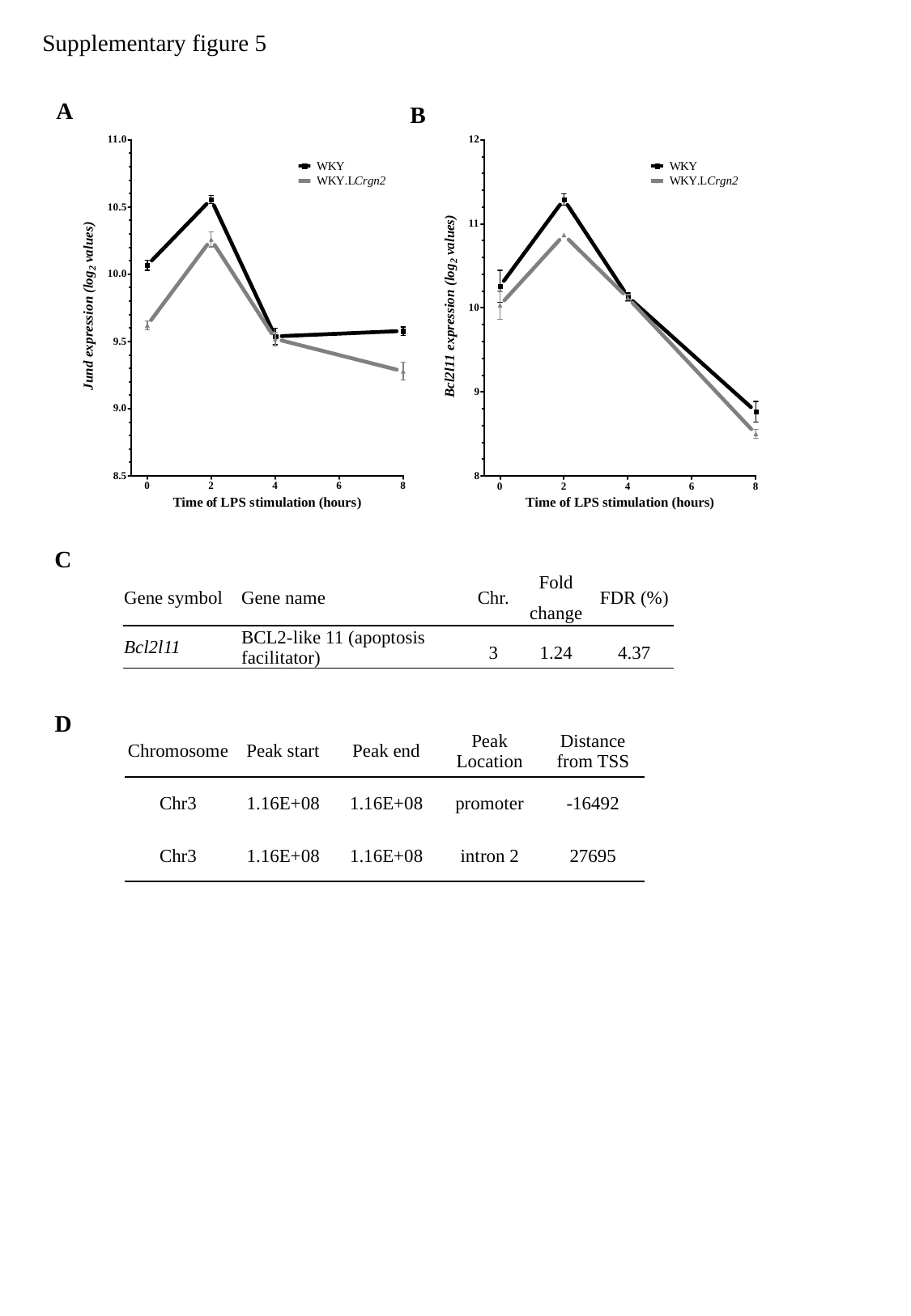

Supplementary figure 5
A
B
C
| Gene symbol | Gene name | Chr. | Fold change | FDR (%) |
| --- | --- | --- | --- | --- |
| Bcl2l11 | BCL2-like 11 (apoptosis facilitator) | 3 | 1.24 | 4.37 |
D
| Chromosome | Peak start | Peak end | Peak Location | Distance from TSS |
| --- | --- | --- | --- | --- |
| Chr3 | 1.16E+08 | 1.16E+08 | promoter | -16492 |
| Chr3 | 1.16E+08 | 1.16E+08 | intron 2 | 27695 |
