# Supplementary material for: Combined ChIP-Seq and transcriptome analysis identifies AP-1/JunD as a primary regulator of oxidative stress and IL-1β synthesis in macrophages
Source: BMC Genomics. 2013 Feb 11;14:92. doi: 10.1186/1471-2164-14-92 (PMC3608227; doi:10.1186/1471-2164-14-92)
Supplement: Additional file 2: Table S1 — Validation of differentially expressed genes identified by siRNA microarray data analysis with quantitative PCR. Table S2. Sequencing and mapping statistics for ChIP-Seq in WKY and WKY.LCrgn2 BMDMs. Table S3. Gene ontology analysis of JunD-bound genes in basal WKY BMDMs. Table S4. Gene ontology analysis of JunD-bound genes in basal WKY.LCrgn2 BMDMs. Table S5. Gene ontology analysis of JunD-bound genes in LPS stimulated WKY.LCrgn2 BMDMs. Table S6. Gene ontology analysis of JunD-bound genes in LPS stimulated WKY BMDMs. Table S7. Sequences of the four individual siRNAs that comprise siGENOME SMARTpool M-092127-00-0010 (Dharmacon). Table S8. Primer sequences used for qRT-PCR validation of microarray data. Table S9. Primer sequences used for qPCR validation of ChIP-Seq data. [file 1471-2164-14-92-S2.docx]

Supplementary Figure 1. Genome-wide expression analysis in basal and LPS stimulated BMDMs

Genome wide expression analysis by microarrays was performed in BMDMs transfected with rat *Jund* or scrambled control siRNA for the unstimulated condition (A) or following eight hours of LPS stimulation (B) in WKY BMDMs and over an eight hour time course of LPS stimulation in WKY and WKY.L*Crgn2* BMDMs (C). Heat maps of hierarchically clustered significantly differentially expressed genes (<5% FDR threshold) are displayed. All experiments were performed in 4 biological replicates for each strain or siRNA transfected.

Supplementary Figure 2. Validation of microarray data between WKY and WKY.L*Crgn2* BMDMs over an eight hour LPS stimulation timecourse.

Validation of microarray data by qRT-PCR. Samples were amplified using a set of four biological replicates with three technical replicates per sample. Relative gene expression was measured by qRT-PCR and normalised with *Hprt* for WKY and WKY.L*Crgn2* BMDMs. *P<0.05; **P<0.01;***P<0.001 statistically significantly different to WKY using a two way ANOVA to compare the overall timecourse with Bonferonni’s post-tests to compare individual time points.

**Supplementary Figure 3. ChIP-Seq peak validations by ChIP-qPCR.**

ChIP-Seq peaks identified at a posterior probability threshold of 0.9 for basal WKY BMDMs were validated by qPCR (**A**) and for LPS stimulated WKY BMDMs (**B**) and WKY.L*Crgn2* BMDMs peaks (**C**). Samples were amplified using a set of biological triplicates with three technical replicates per sample. Results expressed as mean fold change over IgG**.** **P<0.01, *P<0.05, ns; non-significant using a paired t-test (one-tailed) to compare whether % input for the JunD ChIP qPCR was significantly different to % input for IgG.

**Supplementary Figure 4. *Il1b* and *Prkca* confirmed as primary JunD targets by qPCR validation.**

The aligned reads comprising peak passing the posterior probability threshold of 0.9 for each JunD-bound gene in the WKY strain in the basal state for *l1b* (**A**) and the LPS stimulated state for *Prkca* (**B**) are shown in genome browser views along with the peak in the WKY.L*Crgn2* strain. Samples from WKY and WKY.L*Crgn2* strains were amplified using three biological replicates with three technical replicates per sample. Results expressed as mean fold change over IgG. *P<0.05; **P<0.01; using a one-tailed unpaired t-test to detect statistically significant differences between the strain and condition pairs. Error bars represent standard error of the mean.

**Supplementary Figure 5. Integrative analysis identifies the transcription factor *Bcl2l11* as a primary JunD target**

*Jund* microarray-determined expression patterns in WKY and WKY.L*Crgn2* BMDMs over an eight hour LPS timecourse using four biological replicates per strain were used for Spearman correlation analysis (**A**) with the rest of the transcripts on the microarrays. The expression of *Bcl2l11* (**B**) was significantly correlated to the *Jund* expression pattern (Spearman correlation 0.9, corrected p-value=8.6x10^-5^). Significant differential expression of the gene was seen following siRNA knockdown of *Jund* (**C**). Fold changes are of control siRNA versus *Jund* siRNA expression. The positive fold change indicates higher expression in BMDMs transfected with scrambled control siRNA i.e. with a higher level of *Jund* expression compared to *Jund* siRNA. Abbreviations: Chr.; chromosome, FDR: false discovery rate. Three JunD binding events were identified at a posterior probability threshold of 0.9 in LPS stimulated WKY BMDMs (**D**) located in the gene promoter and second intron.

**Supplementary Table 1** **Validation of differentially expressed genes identified by siRNA microarray data analysis with quantitative PCR**


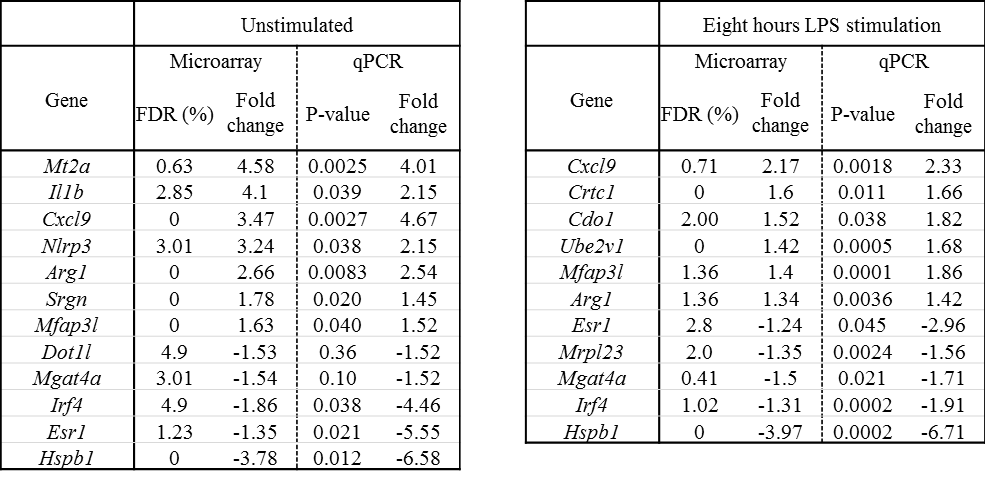


Microarray data for the comparisons between scrambled control and *Jund* siRNA transfected unstimulated and eight hour LPS stimulated BMDMs was validated by qRT-PCR. Microarray FDR % and fold changes are listed for each gene. A positive fold change indicates higher expression in scrambled control siRNA transfected BMDMs i.e. those BMDMs with higher levels of *Jund* expression. qRT-PCR validation was carried out using a set of four biological replicates with three technical amplification replicates per siRNA type. Relative gene expression was normalised to *Hprt* and used to generate fold change values. Positive fold changes indicate upregulated expression in scrambled control transfected BMDMs. A two-tailed unpaired t-test was used to detect statistically significant differences between the WKY and LEW replicates.

Supplementary Table 2 Sequencing and mapping statistics for ChIP-Seq in WKY and WKY.L*Crgn2* BMDMs

| Dataset | Total sequence (megabases) | Total number of uniquely mapped reads | Duplicate reads (%) | Non duplicate unique mapped reads |
| --- | --- | --- | --- | --- |
| WKY basal | 1861.0 | 35,140,250 | 12.55 | 30,730,584 |
| WKY LPS | 1923.9 | 36,721,333 | 15.23 | 31,129223 |
| WKY.L*Crgn2* basal | 2374.9 | 44,915,617 | 8.83 | 40,947698 |
| WKY.L*Crgn2* LPS | 2110.2 | 40,173,323 | 13.95 | 34,569,023 |
|  |  |  |  |  |
| WKY basal input | 1010.0 | 19,217,924 | 1.67 | 18,896,825 |
| WKY LPS input | 772.7 | 14,610,110 | 2.30 | 14,273,377 |
| WKY.L*Crgn2* basal input | 1039.8 | 19,968,619 | 3.37 | 19,296,015 |
| WKY.L*Crgn2* LPS input | 868.1 | 16,959,279 | 11.22 | 15,057,211 |

Total sequence yield per sample lane are shown with numbers of mapped and non-duplicate mapped reads. Samples labelled input represent control chromatin samples not exposed to antibody but subjected to all other processing stages.

Supplementary Table 3 Gene ontology analysis of JunD-bound genes in basal WKY BMDMs

| Gene ontology term (BP_FAT or KEGG pathway) | Genes (n) | Fold Enrichment | Bonferroni corrected P-Value |
| --- | --- | --- | --- |
|  |  |  |  |
| rno05332:Graft-versus-host disease | 27 | 2.94 | 1.43E-05 |
| rno04940:Type I diabetes mellitus | 29 | 2.69 | 5.02E-05 |
| rno04144:Endocytosis | 65 | 1.80 | 1.65E-04 |
| rno05330:Allograft rejection | 26 | 2.73 | 1.71E-04 |
| rno05320:Autoimmune thyroid disease | 26 | 2.37 | 0.0038 |
| rno05416:Viral myocarditis | 33 | 2.11 | 0.0040 |
| rno04514:Cell adhesion molecules (CAMs) | 48 | 1.80 | 0.0051 |
| GO:0007267~cell-cell signalling | 93 | 1.56 | 0.024 |
| GO:0007398~ectoderm development | 41 | 2.03 | 0.027 |
| GO:0030855~epithelial cell differentiation | 40 | 2.04 | 0.034 |
| rno04612:Antigen processing and presentation | 31 | 1.96 | 0.035 |

Enrichment for biological process functional annotation terms and KEGG canonical pathways used a Bonferroni corrected p-value threshold of <0.05. Abbreviations: BP_FAT, subset of Biological Process Gene Ontology (GO) terms generated by DAVID; n, number of involved genes

Supplementary Table 4 Gene ontology analysis of JunD-bound genes in basal WKY.L*Crgn2* BMDMs

| Gene ontology term (BP_FAT or KEGG pathway) | Genes (n) | Fold Enrichment | Bonferroni corrected P-Value |
| --- | --- | --- | --- |
|  |  |  |  |
| rno05332:Graft-versus-host disease | 19 | 4.89 | 2.18E-06 |
| rno05330:Allograft rejection | 19 | 4.70 | 4.38E-06 |
| rno04940:Type I diabetes mellitus | 20 | 4.37 | 6.58E-06 |
| rno05320:Autoimmune thyroid disease | 20 | 4.30 | 8.89E-06 |
| rno04612:Antigen processing and presentation | 24 | 3.58 | 1.39E-05 |
| rno04144:Endocytosis | 36 | 2.35 | 3.59E-04 |
| rno05416:Viral myocarditis | 21 | 3.17 | 8.63E-04 |
| rno04514:Cell adhesion molecules (CAMs) | 28 | 2.48 | 0.0022 |
| GO:0019882~antigen processing and presentation | 21 | 3.40 | 0.0063 |
| GO:0002474~antigen processing and presentation of peptide antigen via MHC class I | 11 | 6.43 | 0.0074 |
| GO:0048002~antigen processing and presentation of peptide antigen | 13 | 4.48 | 0.050 |

Enrichment for biological process functional annotation terms and KEGG canonical pathways used a Bonferroni corrected p-value threshold of <0.05. Abbreviations: BP_FAT, subset of Biological Process Gene Ontology (GO) terms generated by DAVID; n, number of involved genes

Supplementary Table 5 Gene ontology analysis of JunD-bound genes in LPS stimulated WKY.L*Crgn2* BMDMs

| Gene ontology term (BP_FAT or KEGG pathway) | Genes (n) | Fold Enrichment | Bonferroni corrected P-Value |
| --- | --- | --- | --- |
|  |  |  |  |
| GO:0019882~antigen processing and presentation | 18 | 8.71 | 2.66E-08 |
| rno05332:Graft-versus-host disease | 14 | 10.03 | 6.60E-08 |
| rno05330:Allograft rejection | 14 | 9.65 | 1.11E-07 |
| rno04940:Type I diabetes mellitus | 14 | 8.53 | 5.80E-07 |
| rno05320:Autoimmune thyroid disease | 14 | 8.39 | 7.19E-07 |
| rno04612:Antigen processing and presentation | 16 | 6.64 | 1.20E-06 |
| GO:0002474~antigen processing and presentation of peptide antigen via MHC class I | 10 | 17.47 | 3.34E-06 |
| rno04144:Endocytosis | 21 | 3.82 | 4.79E-05 |
| rno05416:Viral myocarditis | 14 | 5.88 | 6.09E-05 |
| rno04514:Cell adhesion molecules (CAMs) | 17 | 4.20 | 2.56E-04 |
| GO:0048002~antigen processing and presentation of peptide antigen | 10 | 10.30 | 6.11E-04 |

Enrichment for biological process functional annotation terms and KEGG canonical pathways used a Bonferroni corrected p-value threshold of <0.05. Abbreviations: BP_FAT, subset of Biological Process Gene Ontology (GO) terms generated by DAVID; n, number of involved genes

Supplementary Table 6 Gene ontology analysis of JunD-bound genes in LPS stimulated WKY BMDMs

| Gene ontology term (BP_FAT or KEGG pathway) | Genes (n) | Fold Enrichment | Bonferroni corrected P-Value |
| --- | --- | --- | --- |
|  |  |  |  |
| GO:0007242~intracellular signalling cascade | 326 | 1.38 | 3.66E-08 |
| GO:0006793~phosphorus metabolic process | 287 | 1.32 | 1.32E-04 |
| GO:0006796~phosphate metabolic process | 286 | 1.32 | 1.58E-04 |
| GO:0007243~protein kinase cascade | 109 | 1.60 | 2.35E-04 |
| GO:0016310~phosphorylation | 244 | 1.34 | 5.67E-04 |
| GO:0006468~protein amino acid phosphorylation | 215 | 1.37 | 6.93E-04 |
| rno04010:MAPK signalling pathway | 104 | 1.47 | 0.0011 |
| GO:0030182~neuron differentiation | 165 | 1.42 | 0.0017 |
| GO:0032989~cellular component morphogenesis | 140 | 1.46 | 0.0020 |
| GO:0000902~cell morphogenesis | 127 | 1.47 | 0.0061 |
| GO:0010604~positive regulation of macromolecule metabolic process | 270 | 1.28 | 0.0064 |
| GO:0010033~response to organic substance | 299 | 1.26 | 0.0075 |
| GO:0030001~metal ion transport | 145 | 1.41 | 0.012 |
| GO:0030030~cell projection organization | 132 | 1.44 | 0.014 |
| GO:0048666~neuron development | 127 | 1.44 | 0.020 |
| GO:0006811~ion transport | 221 | 1.30 | 0.030 |
| rno05332:Graft-versus-host disease | 27 | 1.99 | 0.030 |
| GO:0006928~cell motion | 147 | 1.39 | 0.031 |
| GO:0009719~response to endogenous stimulus | 193 | 1.32 | 0.036 |
| GO:0009611~response to wounding | 149 | 1.38 | 0.037 |

Enrichment for biological process functional annotation terms and KEGG canonical pathways used a Bonferroni corrected p-value threshold of <0.05. Abbreviations: BP_FAT, subset of Biological Process Gene Ontology (GO) terms generated by DAVID; n, number of involved genes

Supplementary Table 7. Sequences of the four individual siRNAs that comprise siGENOME SMARTpool M-092127-00-0010 (Dharmacon)

| ***Jund* siRNA** | **Target sequence** | **Molecular weight (g/mol)** |
| --- | --- | --- |
| D-092127-01 | GAAAGUCAAGACCCUCAAA | 13400.9 |
| D-092127-02 | CAUCGCCGCUUCCAAAUGC | 13418.0 |
| D-092127-03 | GAAAGGCUGAUCAUCCAGU | 13415.9 |
| D-092127-04 | GAAGAAAGACGCGCUGACG | 13446.0 |

Supplementary Table 8. Primer sequences used for qRT-PCR validation of microarray data

| Gene for validation | Forward primer | Reverse primer |
| --- | --- | --- |
| *Arg1* | ACGGCAGTGGCGTTGACCTT | ACAAGCCCTTGGGAGGAGCA |
| *Cables2* | GGGCCTGCGGATCAGTGACC | TCTGCTCCCAGCTCAACGCC |
| *Ccl22* | CCGATGCAGGTCCCTATGGTGCC | AGGCTTGCGGCAGGACTTTGAG |
| *Ccr2* | GGGGCCACCACACCGTATGAC | TACCAGGGAGTAGAGTGGGGGCA |
| *Cdo1* | AGCAATCCTGCCGAGTGGGCT | CGTGAATACTGCTGCCATGCCC |
| *Crim1* | GCCTCAGGGAAGCCGGGAGA | TCCGCTGTGAGCCGCACTTG |
| *Crtc1* | ACTGCACAACCAGAAGCAGGCG | CGACTTCTGCAGTTGAAGCCGC |
| *Cxcl9* | ATCACTGTGGAGTTCGAGGAACCC | GTTGCAGTTAGGGCTTGGGGCA |
| *Cyp2j4* | GATCGAGAATCCATGCCCTA | TCCCTGTGCAGTGCAGTTAG |
| *Dot1l* | GACTTGGCCTGCTGGGCTGG | TCCACGTTTGGGGCAGCACC |
| *Esr1* | CCTTGATCACACACCGCGCCA | CGGATGAGCCACCCTGCTGGT |
| *Gzmb* | TGCTCTAGGACAGATGGCAGCA | GGGTTGTCACAGCCTTGTGGCA |
| *Hprt* | TCTTTGCTGACCTGCTGGATT | TTTTATGTCCCCCGTTGACTG |
| *Hspb1* | CCCGGAAATACACGCTCCCTCC | CGGGCCTCGAAAGTGACCGG |
| *Il10* | AGGCAGAGAACCATGGCCCAGA | GGGAGAAATCGATGACAGCGTCGC |
| *Il1b* | TGCCTCGTGCTGTCTGACCCA | TCCAGCTGCAGGGTGGGTGT |
| *Il1rn* | CTTTTCTGTGTGATGCCCCT | GTGAAGATGGTGTTTGGGCT |
| *Irf4* | ATGGCAACACTGGAAGGGCGG | ATGGCAACACTGAAGGGCGG |
| *Jund* | GCGCAGCTCAAACAGAAAGT | GCACCGAGTCTGCAAAGAGT |
| *Mafb* | GCCCGCGAGAGAGACGCCTA | GGTCGGGACCCGCTACGACT |
| *Mcoln2* | AGCACAGAGCTGCAGTGGCGTC | GGATCTGGCGTCTGGCTCGGTAT |
| *Mfap3l* | GAGTGATGCCCCCTCCCCCA | CGGACAGACCCCTGCTCCGT |
| *Mgat4a* | TGTTCCAGGCGCCGGACCTA | TGTCTGTCGCAGTGTTTGGCA |
| *Mmp7* | CGCAAGGGGAGATCACGGAGAC | AGTGAGCATCTCCGCCGAGGC |
| *Mmp8* | ACCCCACAGATGTCAAAGGCTGA | TGTCACCATGGTCTCTTGAGACGA |
| *Mrpl23* | CCTACGTGCAGCTGGCCCAC | CGTGGGTCGCTGCTTTGCCT |
| *Mt2a* | CAACTGCCGCCTCCATTCGC | AACAGCAGCTTTTCTTGCAGGAGGT |
| *Nlrp3* | GCGCTGCGGACTGACCCATC | TGCTTCAGTCCCACGCACAGC |
| *Plac8* | TGGAGTCTGCCTCTGTGGGACC | ACCCAGGAATGCCGTATCGGGT |
| *Serpinb2* | TCTGCAACGCATGGGCATGGAA | TGCCACAGTGCCCTCCTCGTT |
| *Slit2* | CCCACCGGAATACACAGGCGAA | TCCCTTTGGCGTCAGGATGCA |
| *Srgn* | TCAGTTCAAGGTTATCCTGCTCGGA | GGTCGAACCGTGGTCCTTTCTCC |
| *Tgfb2* | TTCGCAGGTATCGAATGGCACCT | GCAGGAGATGCGGGGTCTTCC |
| *Tlr5* | GCCCAGAGCCGGTGTCTGTC | GAGGTCCTCGGGCCACCTCA |
| *Tnfrsf14* | CTTCAGGCTGGTGCCGTGTGT | ATGGGGCAGCACTCATCCCAA |
| *Ube2v1* | GCCACCACAGGCTCGGGAGTA | GTCGTCCTCCAGACCCCAGCT |
| *Vcam1* | CCACCGCTGAAGACACCGGGA | GTGGGTTCTTTCGGAGCAACGTTG |
| *Vegfa* | TCGCAGTCCGAGCCGGAGAG | GCAGCCTGGGACCACTTGGC |

Supplementary Table 9. Primer sequences used for qPCR validation of ChIP-Seq data

| Gene for validation | Forward primer | Reverse primer |
| --- | --- | --- |
| *Crtc1_1* | CTGGGAGGGTGCCTGCCTGA | GGGGAAACCGAGGCCCAGGT |
| *Crtc1_2* | CAGCCTGGCCATGACTGGGC | TGGCATGGGCCACAGGAGGA |
| *Ctss* | GCATGGTCAAGGGCAGGCAGT | CCAGGGACAACCCCCAAGTGC |
| *Dusp1* | TGCACAGCATATGCACAGTCCCTA | CGGCCTGGCAGTGCACAAACA |
| *Il1b* | GGCCTTTGGCTTCCTGACTTGGAC | ACTCTCCGGCAAGGAAGGGTGT |
| *Irf4* | ATGCTGCACCCCATTGCCCC | GGTGTCGAGAGGCCGGTCCT |
| *Lpcat1* | ACCCTCTGCATCTGAGGTGCCA | TGGTCCGGAAGAGAAGAGCACA |
| *Mafb* | GCCTTCCAGCCTGCGCTTTCA | ATCCGCCTACTCGCTCGCTCA |
| *Mfap3l* | GGGCTTCAGCTACGAGTGGGACT | AGCGTGCAGTGCAGTGCTCATG |
| *Mpzl2* | GATGCTCCCCTCCCCCACAGA | CCGTGTAGACCAGACCGGAGAC |
| *Mrpl23* | ACCATGGCCAAATCGCTCCCG | TCCCACCTTTCTGGTTTCCGATGT |
| *Pfn2* | GGCAGGGTTGCCCTGGTGAC | TTTCAGGAGCGTGCGAGGCG |
| *Prodh2* | CTGGCCCCTTCCCCTGGTGT | AGGCCCCACCATCAAAGCGC |
| *Rest* | CCCGGCTCTCAGGTGGTGCT | TTCCCCGTCTGAGGAGGGCG |
| *Snapc2* | TGACCCTCCCACATCCCACACC | TGCATAACTGGGGTGCCATGCC |
| *Srgn* | TCTGACAGCTTTCCTGTCCAATGC | GACCAAGGTAGTGGCGTGGGG |
| *Sub1* | CGCCAGCCCATGTGGGGATG | TGCAGTCAACACACGCAGTGCA |
| *Tlr5* | GCAGGTGGCCATAGCTCTCATGC | CCTCAAGCCACGCCCGCTAA |
| *Ube2v1* | CCTTGTTGGGCAGAATGGCCCT | CGGGGGCATTGTGAGGTGCA |
